# Phytonanofabrication of iron oxide particles from the *Acacia jacquemontii* plant and their potential application for the removal of brilliant green and Congo red dye from wastewater

**DOI:** 10.3389/fbioe.2023.1319927

**Published:** 2023-11-24

**Authors:** Shreya Patel, Reema Desai, Bhakti Patel, Daoud Ali, Vinars Dawane, Kamlesh Gadhvi, Virendra Kumar Yadav, Nisha Choudhary, Dipak Kumar Sahoo, Ashish Patel

**Affiliations:** ^1^ Department of Life Sciences, Hemchandracharya North Gujarat University, Patan, India; ^2^ Department of Zoology, College of Science, King Saud University, Riyadh, Saudi Arabia; ^3^ Department of Microbiology and Biotechnology, Sardar Vallabhbhai Patel College Mandleshwar, Madhya Pradesh, India; ^4^ Gujarat Forestry Research Foundation, Gandhinagar, India; ^5^ Department of Veterinary Clinical Sciences, College of Veterinary Medicine, Iowa State University, Ames, IA, United States

**Keywords:** iron oxide nanoparticles, phyto-synthesis, magnetic behavior, ethanolic extract, adsorption

## Abstract

Phytonanofabrication is one of the most promising areas that has drawn the attention of scientists worldwide due to its eco-friendly nature and biocompatibility. In the current investigation, we reported the phyto-assisted formation of iron oxide nanoparticles (IONPs) from a rare species of *Acacia (Acacia jacquemontii)*. First, ethanolic extracts of the stem powder were analyzed by high-performance thin-layer chromatography (HPTLC) for the identification of phytochemicals in the stem sections of *Acacia*. Furthermore, IONPs were synthesized by a chemical co-precipitation method by using the stem extract. The phytonanofabricated iron oxide particles were investigated by UV–Vis spectroscopy, Fourier transform infrared (FTIR) spectroscopy, X-ray diffraction (XRD), field emission scanning electron microscopy (FESEM), and Energy-dispersive X-ray spectroscopy (EDS) for elemental analysis. HPTLC confirmed the presence of several phenols and terpenoids in the ethanolic extracts of the stem. UV–Vis spectroscopy exhibited an absorbance peak at 380 nm, indicating the formation of IONPs, while FTIR spectroscopy showed the typical bands for Fe-O in the range of 599–1,000 cm^−1^ in addition to several functional groups of organic molecules at 1,596 cm^−1^, 2,313 cm^−1^, and 3,573 cm^−1^. XRD exhibits the amorphous nature of IONPs with peaks at 30.7, 35.5, and 62.7 nm. The IONPs were spherical-shaped, whose size varies from 10 to 70 nm, as confirmed by FESEM. EDS exhibited the presence of Fe, O, C, and NaCl. Finally, the phytonanofabricated iron oxide particles were utilized for the removal of brilliant green (BG) and Congo red (CR) dye from the aqueous solution. The removal efficiency of BG dye was up to 54.28%, while that of Congo red dye was up to 36.72% in 120 min and 60 min, respectively. Furthermore, the effect of pH and contact time was also assessed on both the dyes, where CR exhibited maximum removal at acidic pH, i.e., 47.5%, while BG showed maximum removal at pH 10, i.e., 76.59%.

## 1 Introduction

Every year, a large amount of dyes are generated in the textile, food and beverage, and other industries (Islam et al., 2023) for meeting the demands of the increasing population. These dye industries discharge the dye effluent into natural water resources, which leads to water pollution (Shan et al., 2023). It gets mixed with pure water, and this dye-contaminated water increases the biological and chemical oxygen demand of the water. Moreover, the water systems get covered with colored dye due to which light cannot penetrate into the deeper parts of the water bodies (Patel et al., 2022). Due to this, the living beings residing in the lower region of the water bodies do not receive the optimal amount of sunlight for their photosynthetic activity. The prolonged effect of this could lead to their death, especially for phytoplankton and algae. Moreover, the consumption of such dye-loaded water by living beings could result in numerous skin-related disorders, skin cancer, and other diseases (Chequer et al., 2013; Al-Tohamy et al., 2022; Saeed et al., 2022). Brilliant green (BG) and Congo red (CR) dyes are widely used in laboratories as an indicator and in textiles. BG dye, a cationic dye, causes skin and eye irritation, vomiting, diarrhea, shortness of breath, coughing, nausea, etc. (Giri et al., 2020). CR dye is an anionic dye with a complex aromatic structure, which makes it stable against oxidizing agents and non-biodegradable, due to which it could persist in the environment for a longer duration (Kataria and Garg, 2017; Giri et al., 2020; Li et al., 2023). It appears deep blue under acidic conditions while turning into deep red at alkaline pH (Zeng et al., 2019; Csillag et al., 2023). Hence, the treatment of industrial wastewater laden with such dyes before discharge is a major concern for researchers and industries (Liu et al., 2008; Li et al., 2022).

So, the remediation of dyes from these water bodies is of utmost importance. The present approaches for dye removal involve coagulation (Lau et al., 2015), flocculation–coagulation (Pillai and Thombre, 2020), sedimentation, adsorption (Modi et al., 2023), filtration (micro, ultra, and nano), advanced oxidation, and ozonation (Dong et al., 2022; Enache et al., 2023), of which adsorption is a convenient and the most preferred technique for the remediation of dyes from wastewater. The utilization of a low-cost adsorbent, along with its environmentally friendly nature, makes it a technically sustainable approach for dye removal (Kataria and Garg, 2017; Jani, 2023).

In the 21st century, nanotechnology and nanoparticles (NPs) have gained huge popularity among investigators worldwide (Pokrajac et al., 2021; Gnanamoorthy et al., 2023). NPs have gained huge attention in the fields of electronics, medicine, and wastewater treatment (Elnouby et al., 2021; Khan et al., 2022; Selvaraj et al., 2022; Zanata et al., 2022). In environmental cleanup, it is widely used as an adsorbent for the remediation of dyes, pesticides, etc., by using metal and metal oxide NPs. The dyes could be eliminated from the wastewater either by simple adsorption or by photocatalytic degradation. There are several adsorbents like alumina, CuO, zeolites (de Gennaro et al., 2020; Murukutti and Jena, 2022), zinc oxide (Balcha et al., 2016; Amar et al., 2021; Hnamte and Pulikkal, 2022; Choudhary et al., 2023b), clay (Hnamte and Pulikkal, 2022), biosorbents (Aman et al., 2018; Jamoussi et al., 2020; Amari et al., 2023), iron oxide nanoparticles (IONPs) (Wang et al., 2020; Wang et al., 2021; Yadav et al., 2023a), and silica (Yadav et al., 2023b), of which magnetic-based IONPs are of huge importance due to their magnetic nature, easy manipulation by an external magnetic field, and recyclable nature (Alex Mbachu et al., 2023). Moreover, their recyclable property reduces the total cost of dye removal and proves economical. The fabrication of IONPs is possible by all three routes, i.e., chemical, physical, and biological (Yilmaz et al., 2022). The chemical routes involve co-precipitation, sol–gel, and hydrothermal, while physical approaches involve laser ablation, ultrasonication, chemical vapor deposition, and physical vapor deposition. The biological approaches involve phyto-assisted synthesis and microbial synthesis (Triphati and Pirzadah, 2023). Phyto-nanofabrication is preferred due to its biocompatible nature, less involvement of chemicals, and eco-friendly nature (Hu et al., 2022; Chahar et al., 2023; Wang et al., 2023). The chemical route is quite a fast and effective approach, but in order to obtain the desired size, it must be capped with a surfactant or capping agent (Elnouby et al., 2021; Wang et al., 2023). So, this step can be minimized by using plant extracts during the synthesis of IONPs, which have several natural phytochemicals that act as capping agents and surfactants (Ali et al., 2021).

From the various pieces of literature, it has been found that, to date, several plants have been used for the synthesis of IONPs, of which some plants were weeds, some had medicinal values, and some were normal crop plants. To date, very limited research has been conducted on one of the rare species of *Acacia,* which also has medicinal values, and fewer attempts have been made for the synthesis of IONPs from *Acacia* (Yazdanian et al., 2022; Zhang et al., 2023). Even though scientists from several parts of the globe have reported different NP syntheses by using other species of *Acacia*, to date, no attempts have been made for the *Acacia jacquemontii-*mediated synthesis of IONPs**.**

*Acacia* is mainly a thorny, xerophytic shrub (small tree) that is characterized by its zig–zag branching pattern (Mohd Farid et al., 2023) and belongs to the subfamily Mimosoideae that can grow up to a height of 15 m or 50 ft (Rather et al., 2015). It has some common names in local languages, such as *“*Chota babool,” “Bhubaonli, or Baonli” (Rahman et al., 2019). It is distributed in the semi-arid regions around the globe, including the deserts of India and Pakistan (Singh and Joshi, 1979; Gaur and Squires, 2018). In India, it is mainly found in some parts of Gujarat and Rajasthan. It has several valuable phytochemicals due to which its bark, stem, and gums have been used most in pharmaceuticals and medicine/ethnomedicine for the treatment of diarrhea and dysentery (Ashour et al., 2022; Atiya et al., 2022). This plant produces a gum-like substance that has a medicinal property widely used as a food substance by local communities (Ashour et al., 2022). Daud et al. recently analyzed the stem extracts of *A. jacquemontii* and assessed their antioxidant activity and hepatoprotective activity*.* This plant is very rarely exploited for any wet or dry laboratory purposes (Daud et al., 2023).

Dana et al. synthesized iron nanoparticles (FeNPs) using extracts from the pods of *Acacia nilotica*. Moreover, they evaluated the potential of the phytonanofabrication of iron particles for the removal of methyl orange (MO) dye from contaminated water (Da’na et al., 2018). Ocheje (2023) also reported the synthesis of IONPs from the leaves of *A. nilotica* and utilized them for the remediation of heavy metals, especially Cr(II), Cd(II), and Pb(II) from aqueous solutions (Ocheje Ameh, 2023).

Here, the first objective was to analyze the ethanolic extracts of the stem of *A. jacquemontii* by high-performance thin-layer chromatography (HPTLC)*.* Furthermore, the stem extracts were utilized for the phytonanofabrication of IONPs through the chemical co-precipitation method. Then, the synthesized IONPs were characterized by Fourier transform infrared (FTIR) spectroscopy, X-ray diffraction (XRD), field emission scanning electron microscopy (FESEM), and UV–Vis spectroscopy. Finally, the potential of the synthesized IONPs as an adsorbent for the removal of brilliant green and Congo red dye from aqueous solutions was assessed by using a batch adsorption technique (Wroblewski et al., 2020). The effect of pH and contact time on the removal percentage of both dyes was also studied.

## 2 Materials and methods

### 2.1 Materials

The chemicals used include ethanol (Shenzhen, China), ferrous sulfate heptahydrate (Merck, Mumbai, India), ferric chloride anhydrous (Merck, Mumbai, India), and sodium hydroxide pellet (Merck, Mumbai, India). All the chemicals were of laboratory grade.

### 2.2 Methods

#### 2.2.1 Collection of the plant material

**FIGURE 1:**
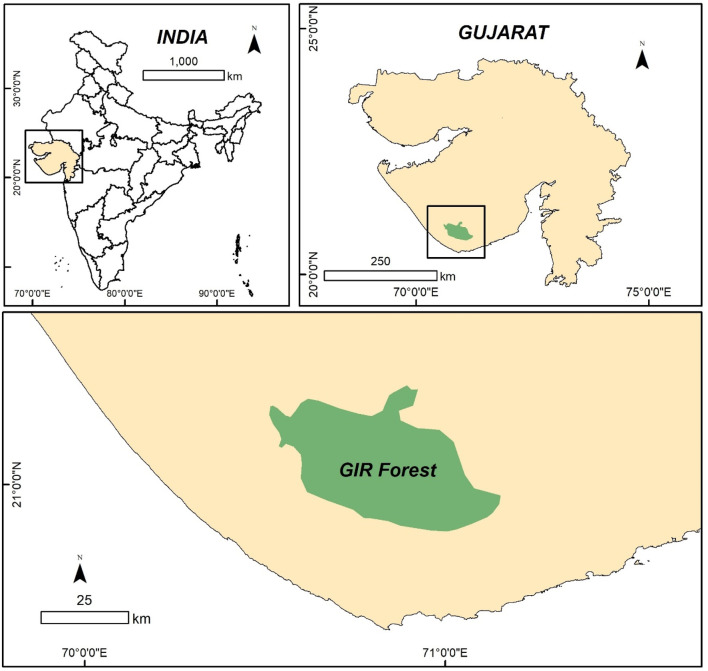
Sampling area: Gir Forest.

The stem of *A. jacquemontii* was collected from the Gir Forest (Gujarat) of the Saurashtra region of Gujarat. The stem of this plant contains lots of thorns, so the thorns were removed, and the collected stem was washed with double distilled water (ddw) to remove fine dust particles. The washed stem was chopped into small fine pieces and dried in the shade in the laboratory. Figure 1 shows the ArcGIS image of the sampling site and collection site, i.e., Gir Forest.

#### 2.2.2 Preparation of ethanolic extracts of *A. jacquemontii*

The collected stem was shade-dried in the laboratory. Furthermore, the dried stem was finely ground using a mortar and pestle to obtain a fine powder. The dried powder sample was mixed with 50 mL ethanol to obtain the ethanolic extracts. Furthermore, the sample was filtered using Whatman grade 1 filter paper and used as a final sample for the HPTLC analysis (Dawane and Pathak, 2020), while the remaining sample was used for the synthesis of IONPs (Ansari et al., 2020).

#### 2.2.3 Synthesis of IONPs using ethanolic extracts of *A. jacquemontii*

**FIGURE 2:**
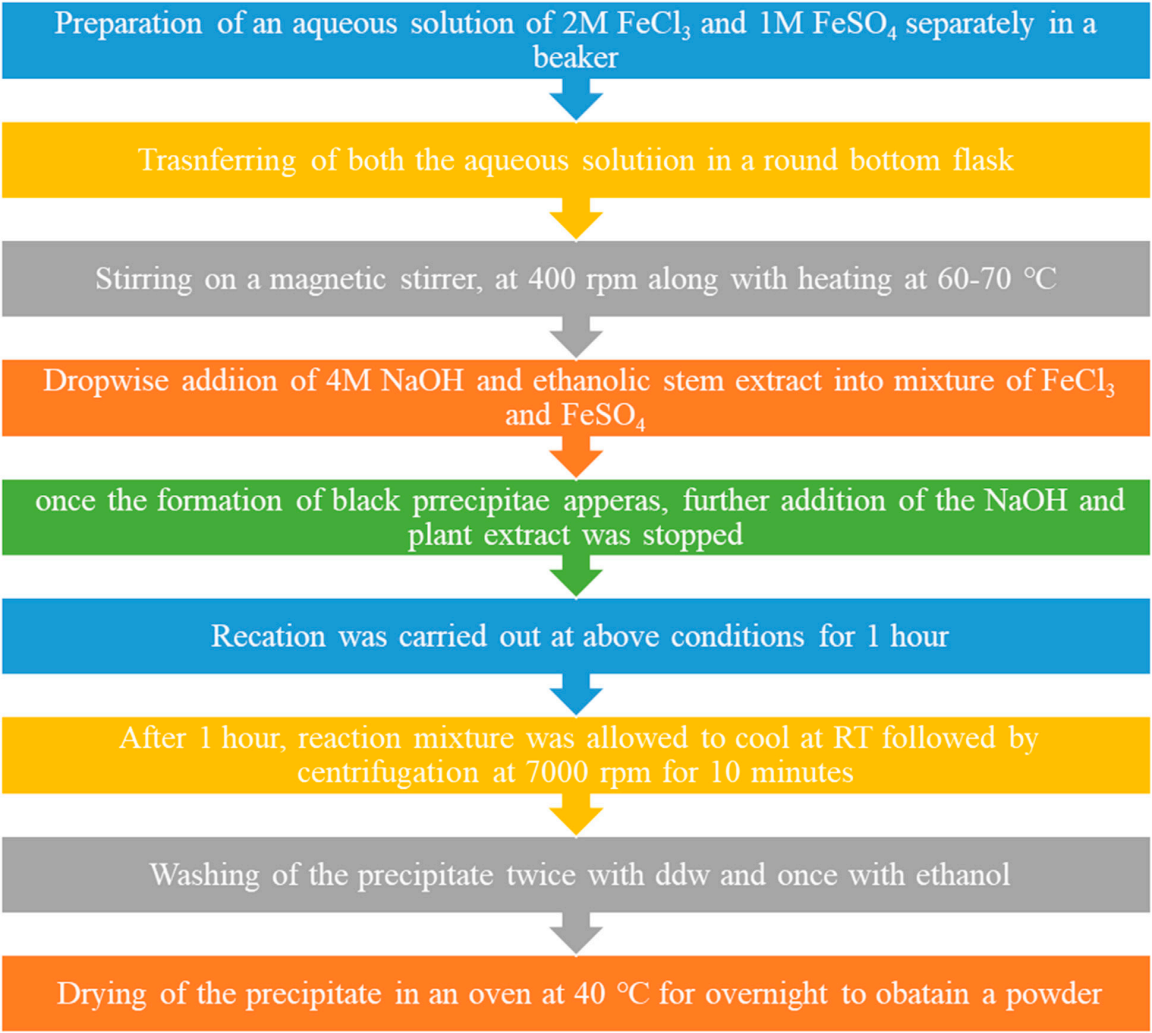
Flowchart for the synthesis of IONPs from the ethanolic extracts of the stem of *A. jacquemontii*.

The plant extract was taken in a separate beaker. Then, both NaOH and plant extract aqueous solutions of 2M FeCl_3_ and 1M FeSO_4_ were prepared in double distilled water and separately in a beaker. A 4M aqueous solution of sodium hydroxide was prepared in a separate beaker. The aqueous solution of ferrous was poured into a round-bottom (RB) flask, which, in turn, was placed on a magnetic stirrer with stirring at 300–400 rpm, along with heating. NaOH was added to the mixture using a dropper into the RB flask. The addition of NaOH was stopped once a black precipitate was formed in the RB flask. Furthermore, the reaction was continued for 1 h at 60°C–70 °C in the RB flask, along with stirring at 400 rpm. After 1 h, the mixture was taken out, cooled, and centrifuged at 7,000 rpm for 5 min. The supernatant was discarded, while the precipitate was retained. The precipitate was washed two times with ddw and once with ethanol. Finally, the precipitate was transferred into a Petri dish and then placed in an oven at 40 °C till it dried completely (Shaibu Auwal et al., 2014; Yadav and Fulekar, 2018). Figure 2 shows a flowchart for the synthesis of IONPs from the ethanolic extracts of the stem of *A. jacquemontii.*

#### 2.2.4 Preparation of brilliant green and Congo red dye aqueous solution

**FIGURE 3:**
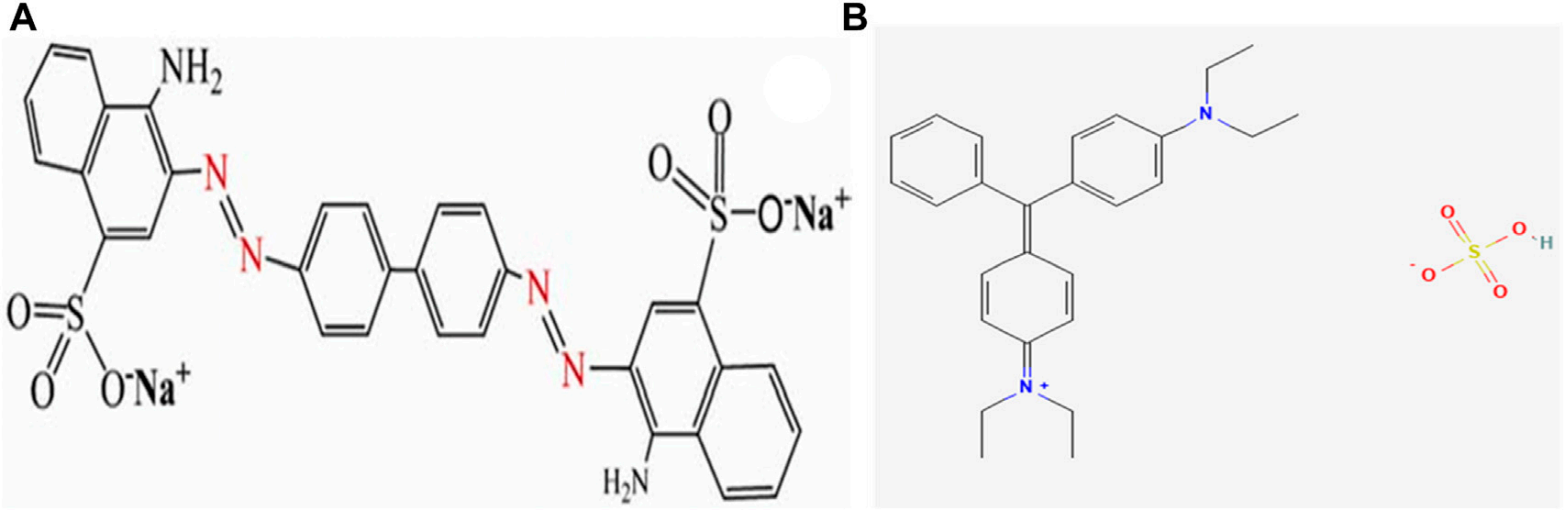
Chemical structure of **(A)** Congo red and **(B)** brilliant green.

An aqueous solution of BG, a cationic dye, and CR, an anionic dye, was prepared by weighing approximately 5 mg of dye into 100 mL of ddw in two separate volumetric flasks to obtain a 50-ppm concentration. Then, the aqueous solution of these dyes was placed on a magnetic stirrer under mild stirring in order to dissolve all the dye granules. Finally, both the dye solutions were filtered through Whatman filter paper grade 42 and stored in a reagent bottle. Figure 3A shows the chemical structure of Congo red, and Figure 3B shows the chemical structure of brilliant green.

#### 2.2.5 Batch adsorption study of brilliant green and Congo red dye removal using IONPs

The remediation of dye was studied by a batch adsorption approach, which was carried out in an incubator shaker at 120 rpm at 30 °C at neutral pH. Approximately 5 mg of IONPs was added to 100 mL of both the aqueous solutions of dyes in two separate Erlenmeyer flasks. Furthermore, an aliquot was taken after every regular interval from 30 min to 120 min. The collected aliquots were then analyzed using a UV–Vis spectrophotometer at a wavelength of 625 nm (λ_625_) for BG and 494 nm for CR (Mariah and Pak, 2020).

#### 2.2.6 Effects of the adsorbent dose on the remediation of dyes

The effect of the dose of the adsorbent on the remediation of dyes was evaluated. Here, the dose of the IONPs was just doubled, i.e., 10 mg in the 100 mL of aqueous solution of the dyes. After adding 10 mg IONPs to both flasks, they were kept in an incubator shaker at 120 rpm at 30 °C for the interaction of the IONPs and dye molecules. Furthermore, an aliquot was taken every 30 min for 120 min and analyzed using a UV–Vis spectrophotometer at a wavelength of 310, 420, and 625 nm and taken at λ_625_ nm for BG and 550 nm for CR.

#### 2.2.7 Effect of pH on the remediation of dyes

The effect of pH on the adsorption of both the dyes was assessed under the aforementioned experimental conditions. The pH of both the dye solutions was adjusted by using 1 mM NaOH and 2% sulfuric acid. Here, four different pHs were used, i.e., 3.4, 6.8, 8.6, and 10. Approximately 100 mL of each dye was prepared at each pH (3.4, 6.8, 8.6, and 10). The initial concentration of both the dyes was kept at 50 mg/L. To all these dye solutions, approximately 10 mg of IONPs was added, and all the flasks were kept in an orbital shaker at 120 rpm at 30 °C for the interaction of the IONPs and dye molecules. Furthermore, an aliquot was taken at 60 min for CR dye and after 120 min for BG dye, and the concentration of the dye was measured by using a UV–Vis spectrophotometer at a wavelength of 625 nm (λ_625_ nm) for BG and 550 nm for CR (Said et al., 2021).

#### 2.2.8 Regeneration study of IONPs

The recycle study of the IONPs was conducted by washing the residual IONPs from the first cycle with 0.1 NaOH, followed by drying at 50 °C for overnight in an oven. Furthermore, the dried and washed IONPs were reused for the remediation of 50 ppm CR dye from the aqueous solutions. An aliquot was taken at 60 min where there was maximum removal percentage and analyzed using a UV–Vis spectrophotometer. Again, the IONPs were recovered, washed with 0.1 NaOH, and finally, dried in an oven at 50 C. Again, IONPs were used to remove 50 ppm dye from the aqueous solutions.

## 3 Characterization of IONPs and phytochemical analysis of the stem of *A. jacquemontii*

### 3.1 HPTLC

The photochemistry profile analysis of the ethanolic extract of *A. jacquemontii* was carried out by using the HPTLC technique according to previously reported methods with slight modifications (Dawane and Pathak, 2020). In brief, the 10 μL ethanolic extract with 8 mm bandwidth was applied on an alumina–silica gel HPTLC plate (Merck, 0.05554), and an n-hexane:ethyl acetate (1:1 v/v) solvent system was used as the plate developmental solvent system. The temperature and relative humidity were kept constant (25.5 +/- 1°C and 67% +/- 2, respectively) throughout the experiment. The developed plate was observed under various electromagnetic radiation zones (before derivatization under UV 254/366 nm and 540 nm as well as after derivatization under 540 nm and UV 366 nm). Anisaldehyde sulfuric acid reagent (ASR) was used as a derivatizing agent. The whole analysis was carried out using CAMAG HPTLC instrumentation (CAMAG semi-automated applicator, CAMAG Visualizer 1, CAMAG Scanner 4, automated derivatized with blue nasal, and CAMAG plate at 110 °C for 5 min).

### 3.2 UV–Vis spectroscopy

UV–visible spectra of IONPs were obtained by dispersing approximately 1 mg of IONPS in ddw and sonicated for 10 min. Furthermore, the UV–Vis measurement was carried out by using a Shimadzu UV-1800 (Japan) UV–Vis spectrophotometer in the range of 200–800 nm at a resolution of 1 nm (Yadav et al., 2023b).

### 3.3 FTIR analysis

FTIR spectra of IONPs synthesized using *A. jacquemontii stems* were obtained by preparing a solid KBr pellet with the sample by maintaining a ratio of 98:2 mg. A pellet was prepared by using a hydraulic pellet-making machine. Furthermore, the IR measurement was carried out to reveal the functional groups where the measurement was carried out in the range of 400–4,000 cm^−1^ at a resolution of 2 nm. The measurement was carried out by using an S6500 Spectrum instrument (PerkinElmer, United States) (Blindheim and Ruwoldt, 2023).

### 3.4 XRD analysis

The XRD patterns of IONPs were recorded using a D8 ADVANCE (Bruker, Netherlands) instrument-equipped accelerometer for revealing the crystalline nature and phase identification of the IONPs. The measurement was carried out in the 2 theta 20–70 with a step size of 0.02 and a time of 5 s per step at a voltage of 40 kV and a current of 30 mA. The crystalline and amorphous phases were determined by XRD.

### 3.5 FESEM analysis for morphological analysis

The size and shape of the phytonanofabricated iron particles were obtained by using the Novo NanoSEM 450 (FEI, United States). The phytonanofabricated iron particles were loaded on the carbon tape, which, in turn, was placed on the aluminum stub holder. The sample was analyzed by the gold sputtering technique before imaging. Imaging was carried out at different magnifications at different scales at 20 kV.

### 3.6 Energy-dispersive X-ray Spectroscopy (EDS) analysis

The elemental investigation of the phyto-synthesized IONPs was carried out by using an Oxford EDS analyzer attached to the FESEM at 20 kV.

## 4 Results and discussion

### 4.1 HPTLC analysis of ethanolic extracts of the *A. jacquemontii* stem

**FIGURE 4:**
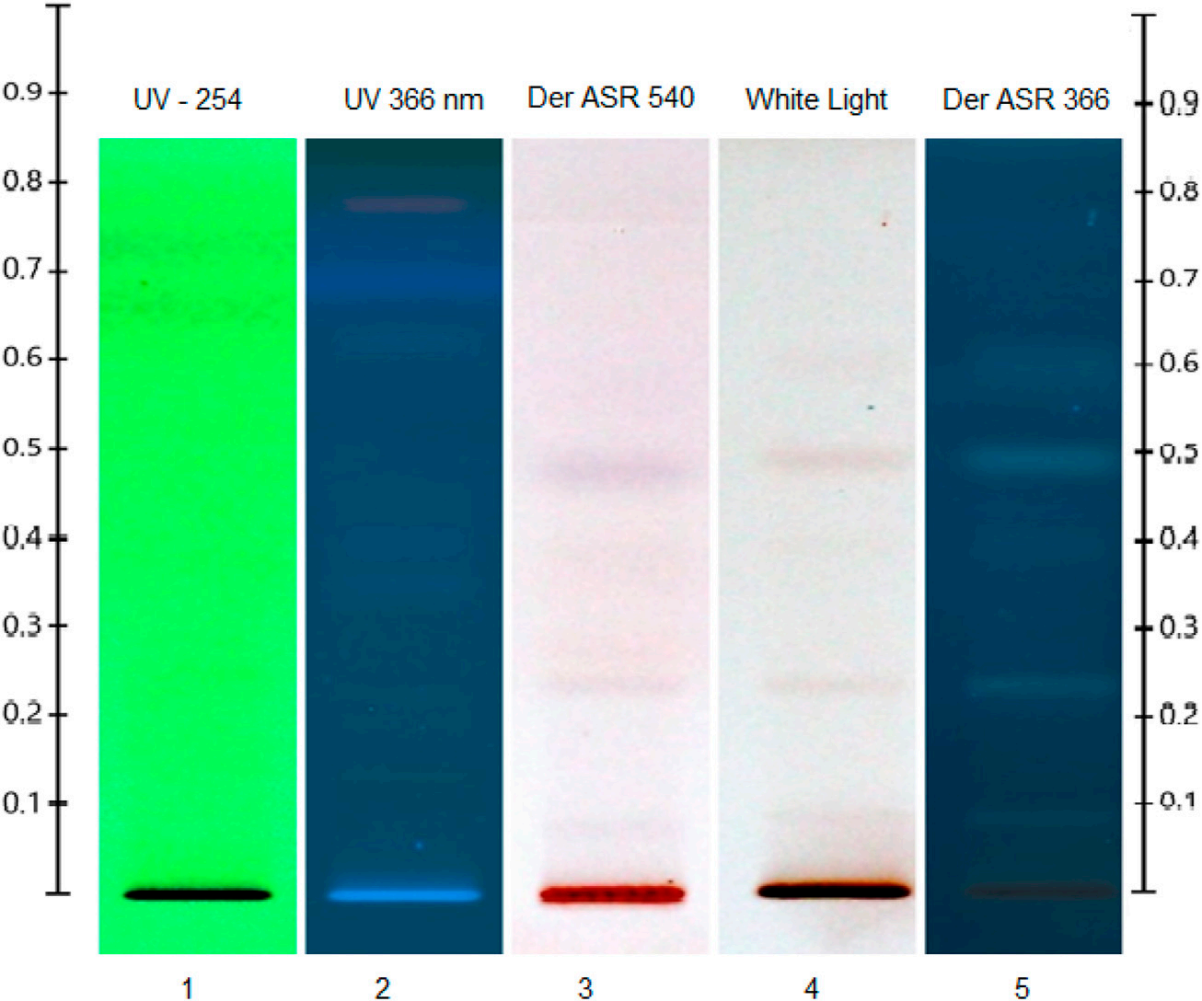
HPTLC chromatogram of ethanolic extracts of the stem of *A. jacquemontii*.

A typical HPTLC chromatogram of the ethanolic extracts of the stem of *A. jacquemontii* is shown in Figure 4. A phytochemical investigation of the plant was carried out in order to obtain basic information about its link with biological activities. The HPTLC investigation revealed the detection of several separate bands respective to various botanical reference materials of primary and secondary phytochemical classes. Thus, separated bands showed detectable signals under various light zones. The dark zones under UV –254 nm, the florescent bands under UV –366 nm, and after the ASR treatment, the similar zones showed violet-to-light/dark-pink zones under white light (540 nm) and the dark zones under UV –366 nm (Reich et al., 2007; Dawane and Pathak, 2020). The developed plate after ASR treatment under white light (Figure 4, white light) and under UV366 nm (Figure 4, UV –366 nm) showed the detection of three major purple-to-violet-colored bands at the Rf max of positions 0.1, 0.25, and 0.48 that showed positive signs for selective phytochemicals, which may be mainly polyphenols or flavonoids or triterpenoidic in nature (Reich et al., 2007; Dawane and Pathak, 2020). The HPTLC results significantly augmented our previous understandings of this plant stem part phytochemical scenario and also followed–interlinked with previous findings with other analytical methods. Ahmed et al. performed a complete phytochemical analysis on this plant species by GC–MS and found approximately 65 types of phytochemicals. Moreover, they further concluded that of these 65 compounds, the major compound was terpenoid (39.28%). Srivastava et al. studied the phytochemicals of dried pods of *A. nilotica,* where they found carbohydrates, proteins, tannins, saponins, starch, phenols, flavonoids, and steroids. These dried pods of *A. nilotica* have 12%–19% tannin, and its quantity increased up to 19%–27% in deseeded pods, and crude proteins were approximately 15.8%. Among polyphenolic compounds, it was reported to have gallic acid, gallolylated flaven-3, chlorogenic acid, m-digallic acid, (+)-catechin, 4-diol, and robidandiol, 3′,4,5′-tetrahydroxyflavan-3,4-diol (Mansi et al., 2021).

### 4.2 Mechanism of formation of IONPs by stem extracts of *A. jacquemontii*

Even though the exact mechanism of the formation of IONPs by plants is not yet well understood, it is assumed that the phytochemicals present in the plant extracts, for instance, alkaloids, polyphenols, flavonoids, and terpenoids, play a crucial role in the reduction of Fe^2+^ ions and the formation of IONPs. These phytochemicals act as a capping, reducing, and stabilizing agent for the developing IONPs. During this process, there is a reduction of iron ions in IONPs, where the plant extracts act as a reducing agent. Furthermore, these iron oxide particles seed and undergo nucleation and aggregation. Then, the developed IONPs are capped by phytochemicals and stabilized. Khan et al. reported the phytonanofabrication of IONPs from *Mentha spicata* using the green synthesis method (Khan et al., 2022).

### 4.3 UV–Vis measurement for preliminary confirmation of IONP formation

**FIGURE 5:**
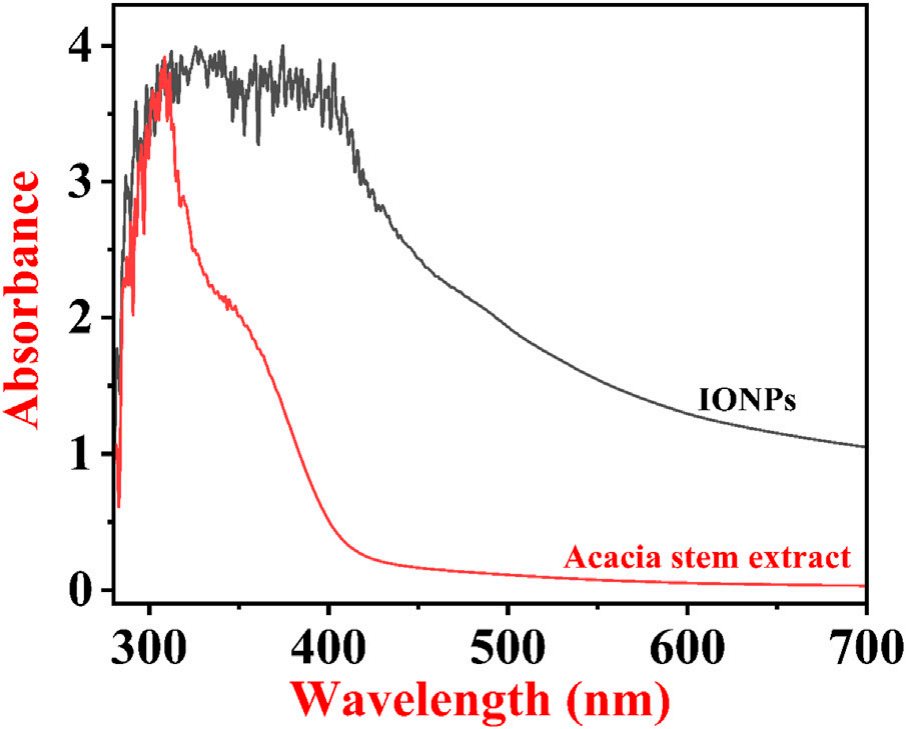
UV–Vis spectra of phytonanofabricated IONPs by the *A. jacquemontii* plant.

Figure 5 shows the typical UV–Vis spectra of ethanolic extracts of the stem of *A. jacquemontii* and IONPs developed from the ethanolic extracts of *A. jacquemontii.* This technique helps in the preliminary confirmation of the formation of IONPs. Initially, there was no absorbance peak in the plant extract for IONPs, while the UV–Vis spectra of IONPs show an absorbance peak at 380 nm, suggesting the development of IONPs (Alangari et al., 2022). Previously, investigators obtained an absorbance peak for the synthesized IONPs in the range of 250–300 nm (Da’na et al., 2018). Mane et al. obtained absorbance peaks at 288 and 420 nm (Mane-Gavade et al., 2021). Kanagasubbulakshmi et al. obtained an absorbance peak at 658 nm for the IONPs fabricated using *Lagenaria siceraria* (Kanagasubbulakshmi and Kadirvelu, 2017). Khan et al. obtained an absorbance peak at 272 nm and a band gap of 2.23 eV for the IONPs synthesized from *M. spicata* (Khan et al., 2022).

### 4.4 FTIR analysis of *A. jacquemontii* stem extracts and the synthesized IONPs for the identification of the functional groups

**FIGURE 6:**
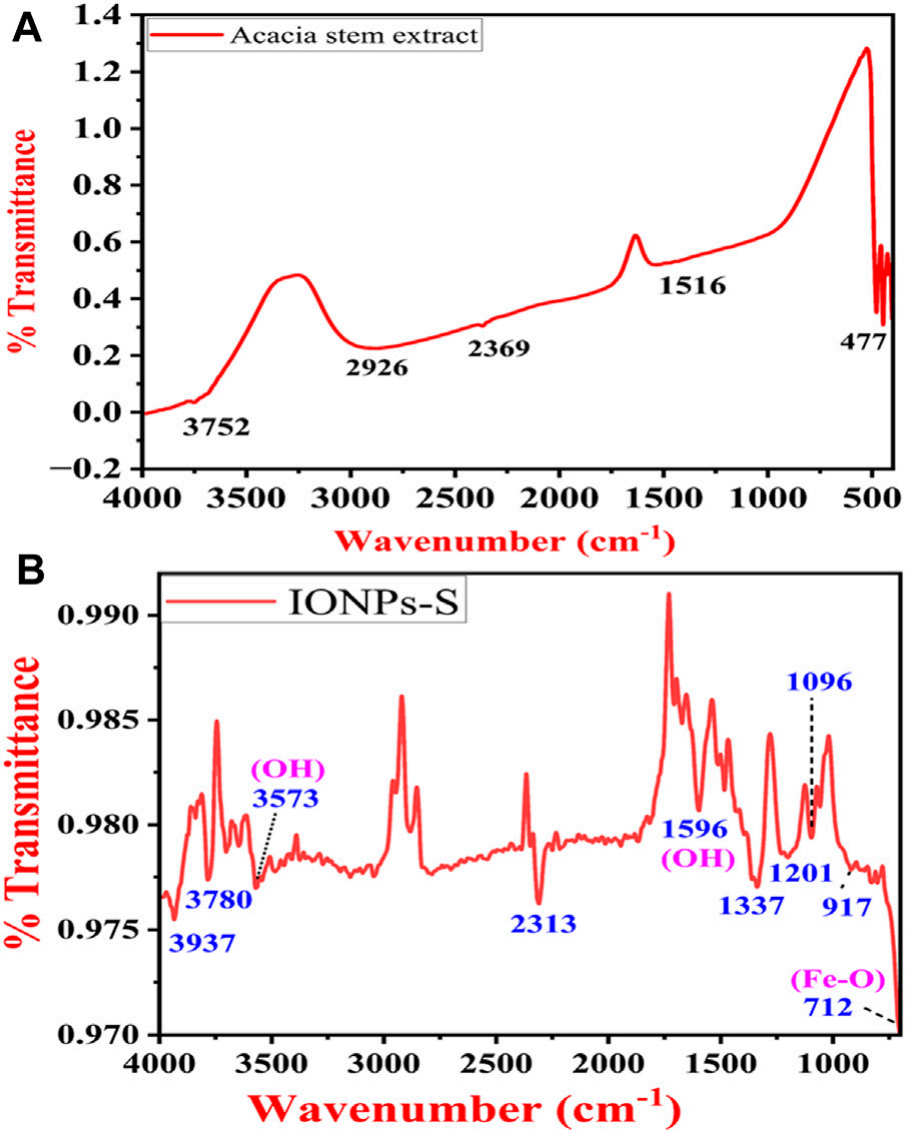
FTIR spectra of the *A. jacquemontii* ethanolic extract **(A).** IONPs synthesized by the *A. jacquemontii* plant **(B)**.

Typical FTIR spectra of the ethanolic extract of *A. jacquemontii* stem and IONPs synthesized using the *A. jacquemontii* plant are shown in Figure 6A,B. The stem extract shows bands at 477, 1,516, 2,369, 2,926, and 3,752 cm^−1^. The band at 1,516 cm^−1^ is attributed to the OH group, while the band at 3,752 cm^−1^ is due to the OH group. Yadav and Fulekar also reported IONP synthesis by using the *Tridax* plant, whose results were in close agreement (Yadav and Fulekar, 2018). Majumdar et al. also obtained bands for the ethanolic extracts for the *A. nilotica* plant at 1,355.4, 1,446.7, 1,528.6, 1,611.4, 1,712.2, 2,853.4, 2,929, and 3,371.8 cm^−1^. They suggested that a broad band at 3,371 cm^−1^ is due to the stretching vibration of the aliphatic and aromatic -OH groups. The bands in the range 1,611–1,400 cm^−1^ are attributed to the presence of aromatic rings in the extract. The band at 1,355 cm^−1^ is attributed to the in-plane bending of OH groups (Majumdar et al., 2013).

The band at 712 cm^−1^ and 917 cm^−1^ is assigned to the Fe-O stretching (Mane-Gavade et al., 2021). It has bands at 1,096 cm^−1^, 1,201 cm^−1^, 1,337 cm^−1^, and 1,596 cm^−1^ (Kanagasubbulakshmi and Kadirvelu, 2017). The band at 1,596 is assigned to the OH group of water molecules present in the sample. Moreover, it could also be attributed to amide C=O stretching, indicating the presence of the –COOH group. In addition to this, it has several bands in the region of 3,500–4,000 cm^−1^, which is attributed to the OH group (Yadav and Fulekar, 2018; Das et al., 2022).

Ocheje Ameh (2023) obtained bands for the IONPs synthesized using the extracts of *A. nilotica* at 602.77 cm^−1^, 715.61 cm^−1^, 1,054.13 cm^−1^, 1,268.24 cm^−1^, 1,631.83 cm^−1^, 2,923.22 cm^−1^, and 3,394.83 cm^−1^, where the first band was assigned to Fe-O stretching. The band at 715.61 cm^−1^ was assigned to the = CH out of the plane, while the band at 1,054.13 cm^−1^ (C-O stretch), 1,268.24 cm^−1^ (C-H wag), and 1,631.83 cm^−1^ (C=O stretch) indicates the association of amide C=O stretching in the reduction process. The band at 2,923.22 cm^−1^ is attributed to C-H aliphatic stretching, and the band at 3,394.83 cm^−1^ is assigned to O-H stretching (Ocheje Ameh, 2023).

### 4.5 Phase identification of IONPs by XRD

**FIGURE 7:**
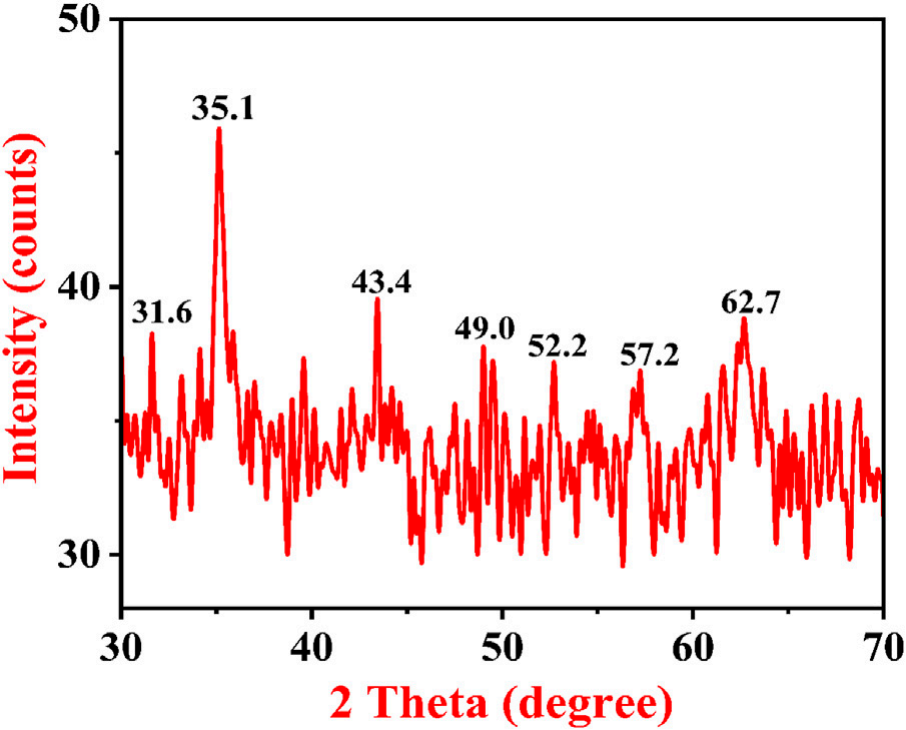
XRD pattern of IONPs synthesized by the *A. jacquemontii* plant.

A typical XRD pattern of *A. jacquemontii* plant-mediated synthesized IONPs is shown in Figure 7. XRD investigation was conducted to identify the crystalline nature of IONPs. The characteristic peaks of iron oxide were obtained at 31.6, 35.1, 43.4, 49.0, 52.2, 57.2, and 62.7°. The peak at 35.1 indicates the maghemite phase of IONPs, which has a reflectance peak at 62.7. The results obtained here were in close agreement with the diffraction peaks obtained for the maghemite phase of IONPs synthesized from the *Tridax* plant by Yadav and Fulekar (2018). Dana (2023) obtained XRD peaks at 2θ = 30°, 36°, 43°,54°, 57°, and 63° with the phase plane of (220), (311), (400), (422), (511). The investigators reported that the average particle size was approximately 11 nm and crystalline in nature (Da’na et al., 2018). Apriandanua et al. also showed similar experimental results for pure and composite forms of ferrous in several investigations (Apriandanu et al., 2023b; 2023a; 2023c).

### 4.6 Morphological analysis of IONPs by FESEM

**FIGURE 8:**
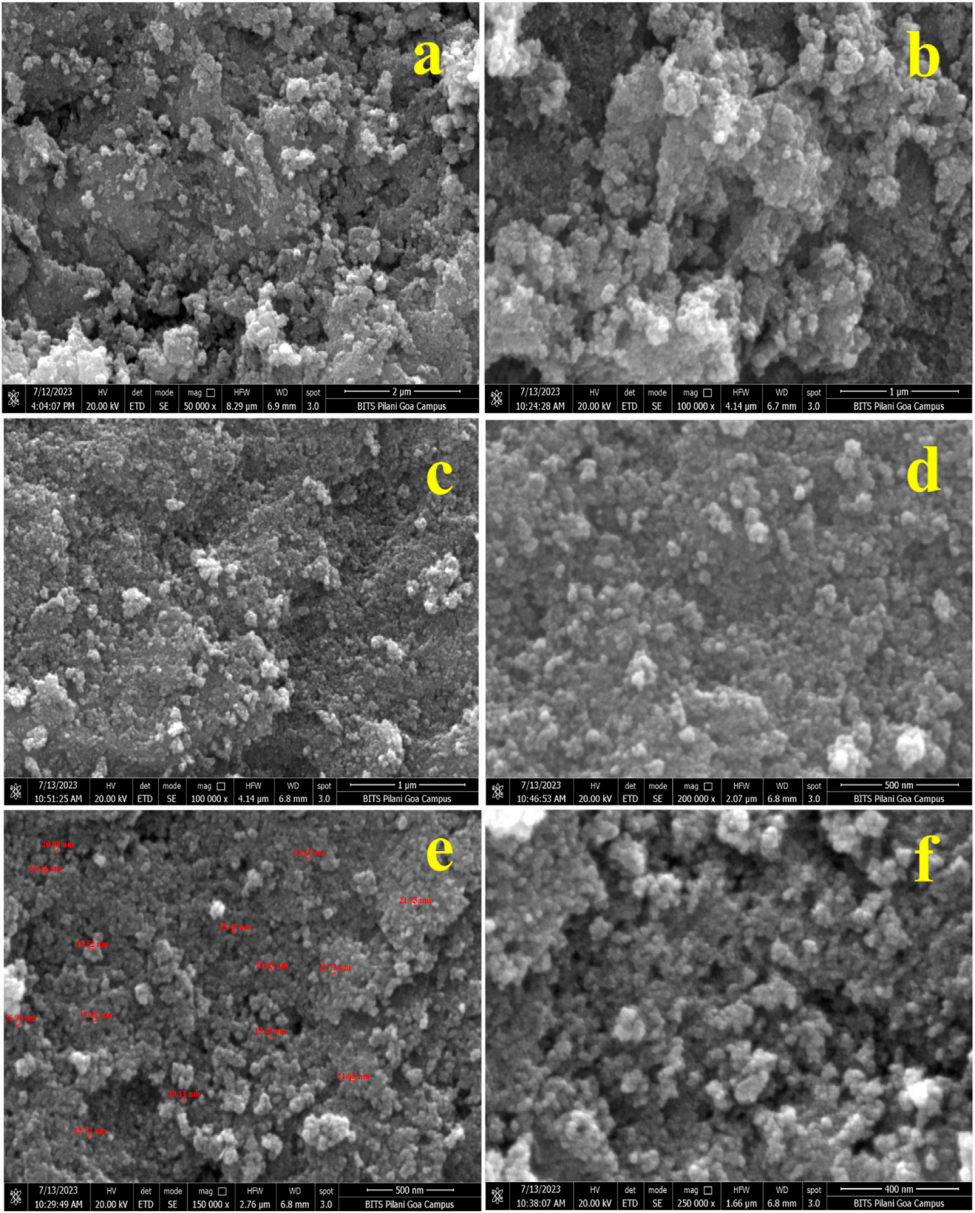
FESEM micrographs of IONPs at different magnifications synthesized by the *A. jacquemontii* plant **(A–F)**.

Figures 8A–F show the FESEM micrographs of IONPs synthesized from the *A. jacquemontii* plant at different magnifications. The IONPs are mainly spherical in shape and aggregated together to form a lump whose size varies from 12 to 26.74 nm. IONPs are found to be agglomerated to form a lump-like structure. Iron oxide in the range of 12.72 nm–26.74 nm has been observed for synthesizing from the *A. jacquemontii* plant stem. Pallela et al. obtained 10–22-nm-sized IONPs with an average size of 16 nm (Pallela et al., 2019). More investigations were conducted with iron oxide in the range of 30 nm–100 nm (Kanagasubbulakshmi and Kadirvelu, 2017).

Dana (2023) also synthesized IONPs by using extracts of dry powder of *A. nilotica*. The obtained particle was mainly spherical in shape and showed high aggregation (Da’na et al., 2018).

### 4.7 Elemental analysis of IONPs by EDS

**FIGURE 9:**
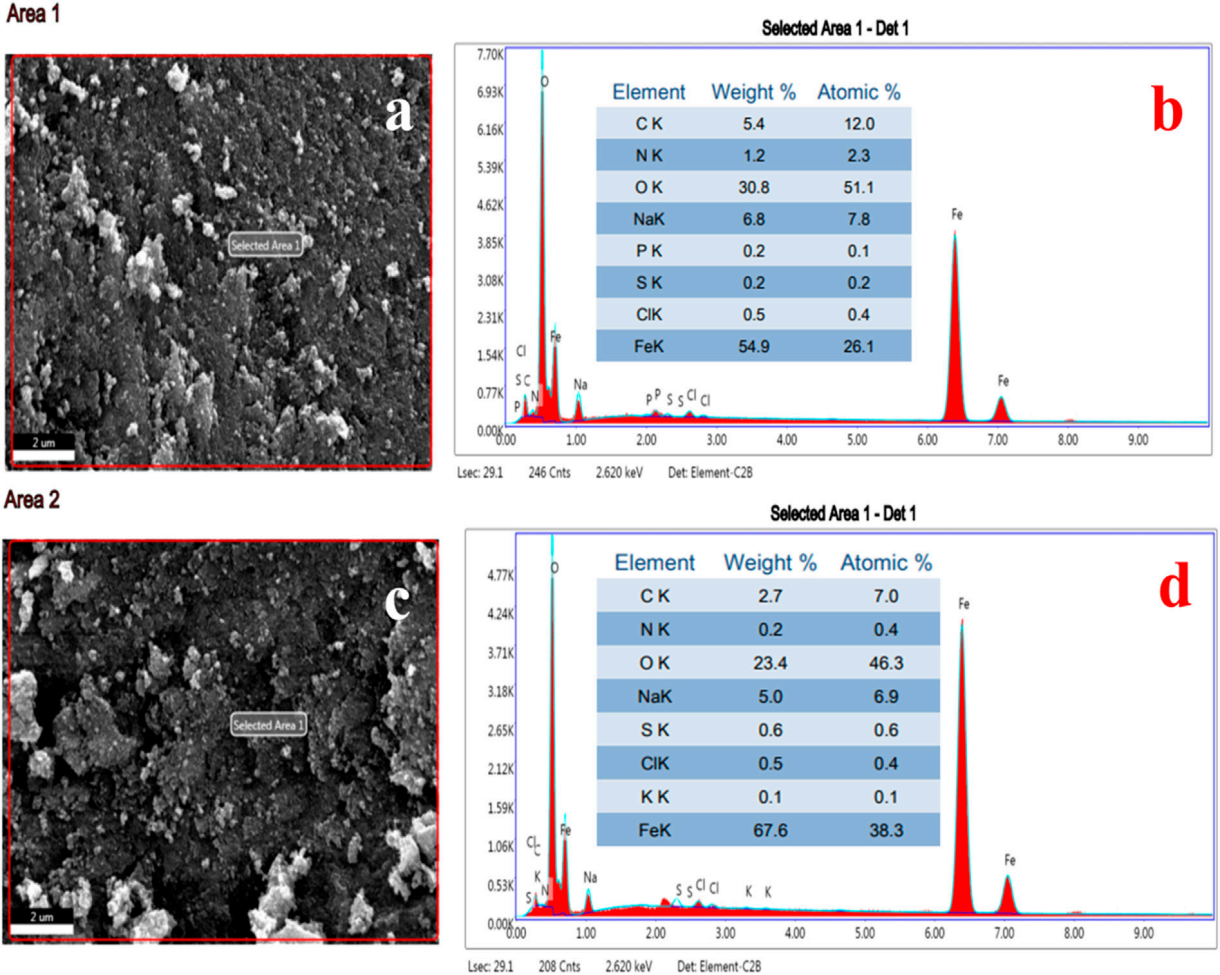
EDS spot **(A, C)**, EDS spectra and elemental table **(B, D)** of IONPs synthesized by *A. jacquemontii* plant.

The EDS spot (Figures 9A,C) and EDS spectra (Figures 9B,D), along with the elemental table, are shown in Figures 9A–D for the IONPs synthesized from the *A. jacquemontii* plant. The elemental composition of the synthesized IONPs was revealed by EDS analysis. The spectra show the peaks of Fe, C, N, O, Na, P, S, and Cl. Out of all elements, the major elements were mainly Fe (54.9 wt%), O (30.8 wt%), Na (6.8 wt%), C (5.4 wt%), N (wt%), Cl (0.5 wt%), P (0.2 wt%), and S (0.2 wt%) from EDS spectra (Figure 9B).

Furthermore, from the EDS spectra (Figure 9D), the results indicated that NPs show spectra of Fe, C, N, O, Na, P, S, and Cl. Out of all elements, the major elements were mainly Fe (67.8 wt%), O (23.4 wt%), Na (5.0 wt%), C (2.7 wt%), S (0.6 wt%), Cl (0.5 wt%), N (0.2 wt%), and K (0.1 wt%). The high percentage of carbon indicates the association of biological molecules from the plant extract with the IONPs. Moreover, P, S, and N also come from the plant extract as all these elements are present in the plant extract. Moreover, most of the chemical precursors used here were mainly laboratory grade and have several impurities. The high percentage of Na and Cl is due to improper washing with ddw and ethanol. All these might be the sources of impurities in the synthesized IONPs.

Pallela et al. obtained 39.37 (wt%) and 60.63 (wt%) oxygen after EDS analysis of the IONPs synthesized from the plant extract of *Sida cordifolia* (Pallela et al., 2019). Dana (2023) also performed EDS analysis of the IONPs synthesized using extracts of *A. nilotica*. They obtained peaks for Fe, O, C, and S, of which Fe was approximately 46.8%, O was 36.47%, and traces of C and S were present. Carbon indicates the presence of organic compounds, along with the synthesized FeO (Da’na et al., 2018; Chen et al., 2020a).

### 4.8 Remediation study of brilliant green and Congo red dye by using IONPs synthesized from the *A. jacquemontii* plant

**FIGURE 10:**
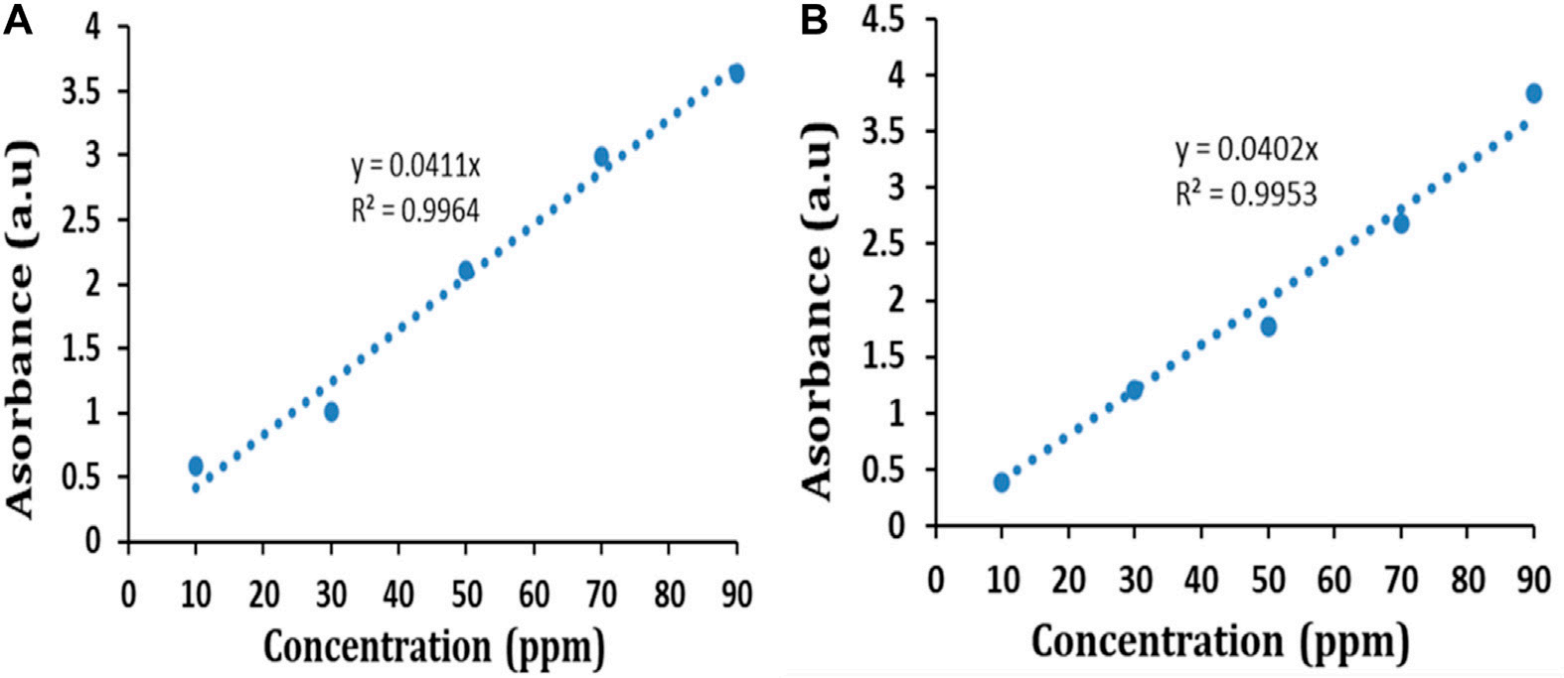
Calibration curve of **(A)** BG dye and **(B)** CR dye.

BG and CR dye adsorption by IONPs was studied by estimating the absorbance at 624 nm and 498 nm, respectively. The initial concentrations of both the dyes in their respective aqueous solutions were calculated based on calibration curves (Figure 10) plotted by taking different known concentrations of each dye compound.

The percent removal of both the dyes at a particular time interval was estimated as follows (Yadav et al., 2023a):

$$\%\;Dye\;removal=\frac{Co-Ct}{Co}\times 100,$$ (1)

where C_o_ = initial dye concentration and C_t_ = dye concentration at a specific time.

**FIGURE 11:**
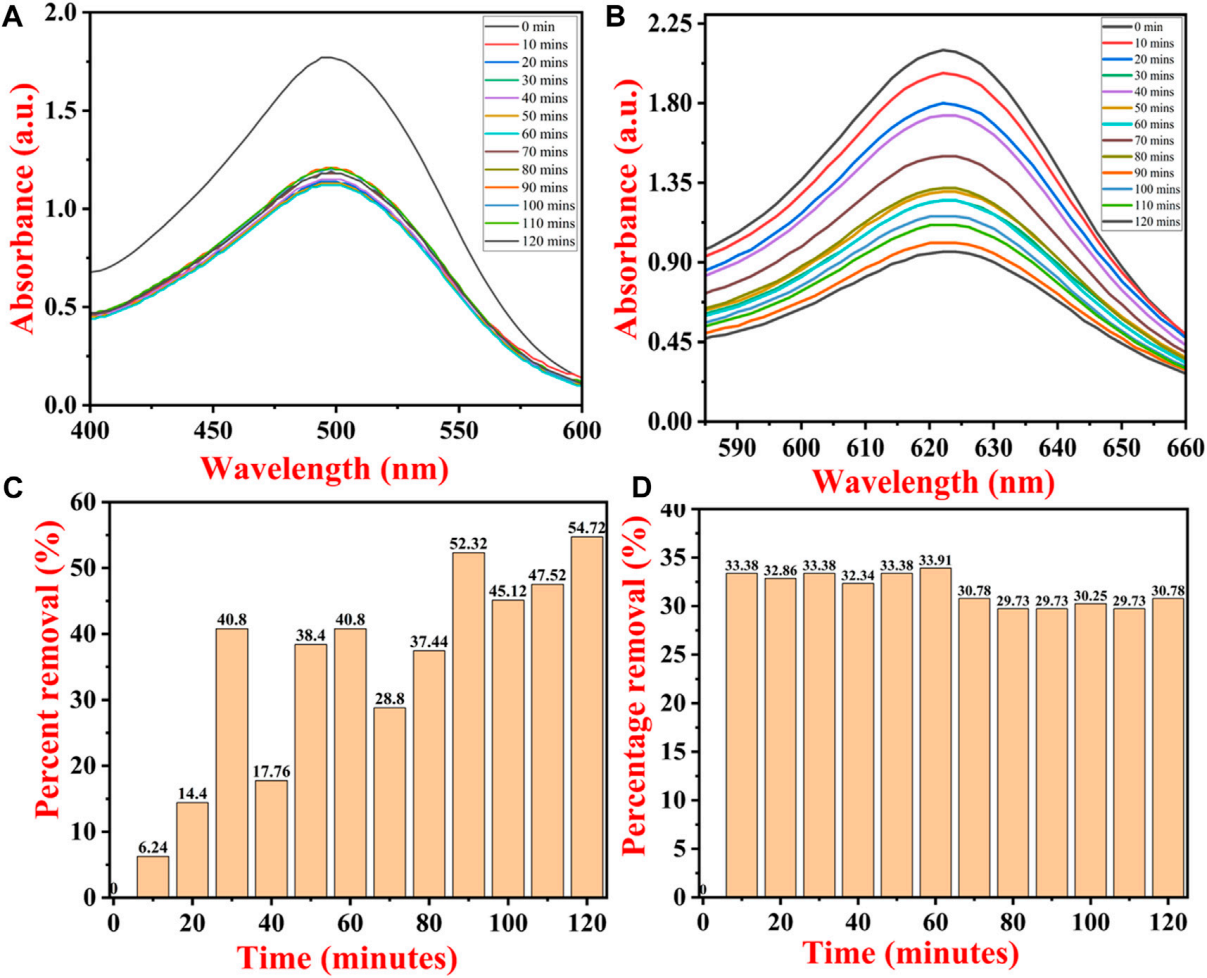
UV–Vis spectra of dye removal with contact time of **(A)** Congo red dye and **(B)** brilliant green dye. Percentage removal of **(C)** BG dye and **(D)** CR dye by IONPs.

Figures 11A,B show the UV–Vis spectra of CR and BG dye removal from the aqueous solutions, along with contact time. Figures 11C,D show the percent removal of both dyes at a specific time interval. The percent removal graph (Figure 11A) shows continuous adsorption and desorption of BG dye. The removal percentage of BG dye by IONPs reached up to 6.24% at 10 min, 14.4% at 20 min, 40.80% at 30 min, and 17.76% at 40 min, and a maximum removal of 54.72% was noted after 120 min.

The initial rate of adsorption was quite high, which was followed by continuous adsorption and desorption of BG dye. The initial higher rate of adsorption onto the IONP surface is due to freely available empty adsorption sites. CR dye was removed up to 33.38% within 10 min, and the maximum removal percentage was observed at 60 min, i.e., 33.91% (Figure 11B). CR attained equilibrium within 10 min, and there were no major changes observed in dye concentration after 10 min as all the adsorption sites on the IONP surface became saturated with CR adsorption.

**FIGURE 12:**
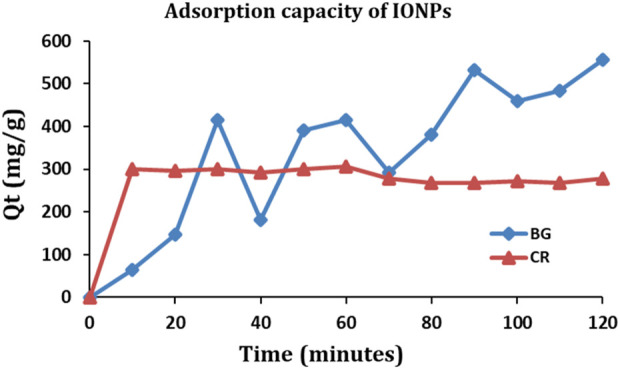
Adsorption capacity of IONPs for BG and CR dye.

The IONPs were examined for their maximum adsorption capacity against BG and CR dye (Figure 12). The maximum adsorption capacity of IONPs for the BG dye was 556.23 mg/g and for CR dye was 305.59 mg/g. The adsorption capacity (Qt) of IONPs for BG and CR dye was also evaluated as follows:

$$Qt=\frac{\left(Co-Ct\right)V}{M},$$ (2)

where Q_t_ = amount of BG and CR dye adsorbed per unit mass of IONPs (mg/g), C_o_ = initial dye concentration and C_t_ = dye concentration at a specific time (t), V = volume of the BG and CR dye in liters (L), and M = mass of IONPs in grams.

### 4.9 Adsorption kinetic study of BG and CR dyes

The adsorption kinetics is applied to evaluate the dynamics and mechanism of adsorption. Pseudo-first-order (PFO) and pseudo-second-order (PSO) kinetics models were prepared by using the derivations provided in Eqs (3) and (4), respectively, to understand the rate of adsorption of BG and CR dye compounds on the surface of IONPs.

$$\ln\left(q_{e}-q_{t}\right)=-kT+\ln\left(q_{e}\right),$$ (3)

$$\frac{t}{qt}=\frac{t}{qe}+\frac{1}{k_{2}qe^{2}},$$ (4)

where q_t_ = amount of BG and CR dye adsorbed per unit mass of IONPs (mg/g), q_e_ = amount of BG and CR dye adsorbed at equilibrium per unit mass of IONPs (mg/g), K_1_ = rate constant for pseudo-first-order kinetics (1/min), K_2_ = rate constant for pseudo-second-order kinetics (g/min/mg), and t = time (min).

**FIGURE 13:**
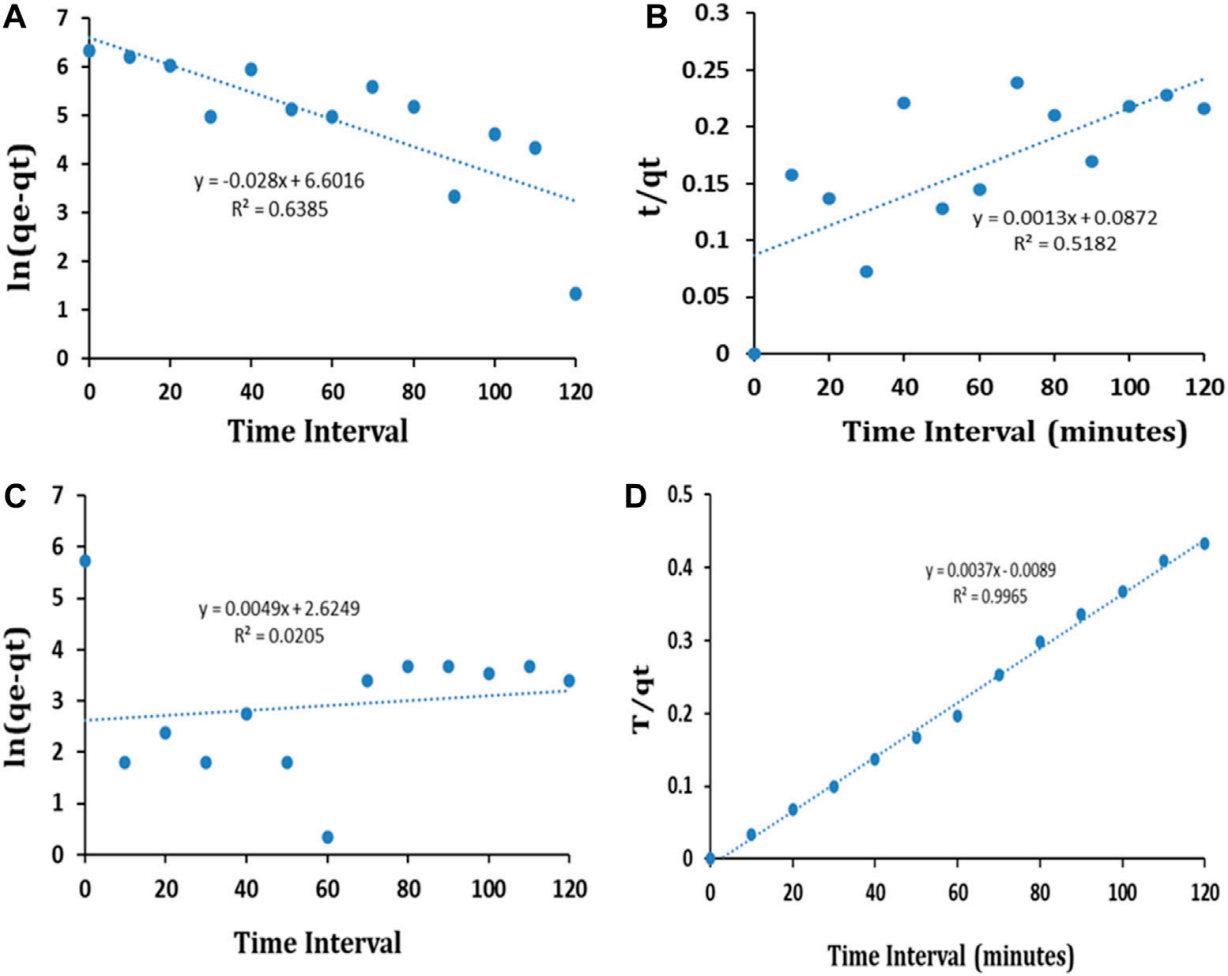
PFO **(A)** and PSO **(B)** kinetic model fit for BG dye and PFO **(C)** and PSO **(D)** kinetic model fit for CR dye.

The kinetic curves for the PFO and PSO kinetic models and the kinetic parameters are shown in Figure 13 and Table 1, respectively.

**TABLE 1:** Kinetics parameters for the removal of BG and CR dye using IONPs.

Dye	C_o_ (mg/L)	q_e_ (mg/g)	Pseudo-first-order		Pseudo-second-order	
			K_1_(min^−1^)	R^2^	K_2_	R^2^
BG	50	560	0.028	0.6385	0.0013	0.5182
CR	50	307	0.0049	0.0205	0.0037	0.9965

The results exhibited that the coefficients of determination (R^2^) for the remediation of BG and CR dye by IONPs were 0.6385 and 0.0205, respectively, using the PFO kinetic model, while 0.9836 and 0.9185 were obtained for the PSO kinetic model (Table 1). It is evident that the PSO kinetic model exhibited a better fitting to the experimental data than the PFO kinetic model.

Here, IONPs were used to remediate the BG and CR dyes from the aqueous solutions. An aliquot of approximately 2–3 mL was collected at a fixed interval of time, which was further analyzed using the UV–Vis spectrophotometer. Using IONPs, the BG dye was removed up to 6.19% at 10 min, 14.28% at 20 min, 40.47% at 30 min, 17.61% at 40 min, 38.09% at 50 min, 40.47% at 60 min, 28.57 at 70 min, 37.14% at 80 min, 51.90% at 90 min, 44.76 at 100 min, 47.14% at 110 min, and 54.28% at 120 min. From the aforementioned reading, it was found that the concentration of BG dye continuously decreased from 10 min to 120 min. The maximum percent removal of BG dye was approximately 54.28% at 120 min. Initially, the adsorption of the dye was low, but as the time periods increased, the adsorption of dye increased.

Similarly, the adsorption of CR dye from the aqueous solution by using IONPs as an adsorbent was analyzed. The removal (%) of CR dye was approximately 6.15% at 10 min, 35.59% at 20 min, 36.15% at 30 min, 35.02% at 40 min, 36.15% at 50 min, 36.72% at 60 min, 33.33% at 70 min, 32.20% at 80 min, 32.20% at 90 min, 32.76% at 100 min, 32.20% at 110 min, and 33.33% at 120 min. The maximum removal (%) of CR dye was approximately 36.15% at 30 min, after which its value decreased gradually and reached up to 33.33% at 120 min. This could be due to the reason that after reaching equilibrium at 30 min, all the adsorption sites must be occupied and desorption of dye molecules might have started. The adsorption increased from 0 to 60 min and then decreased.

Dana et al. also used iron NPs synthesized from *A. nilotica* and applied them for the remediation of 30, 20, 10, and 5 ppm methyl orange dyes from the aqueous solution. They used approximately 0.01 g of the nano adsorbent at 140 rpm, and the experiment was carried out for 24 h. Moreover, they also assessed the effect of pH on the removal of MO dye from the liquid solutions. Furthermore, they also performed a kinetic experiment using approximately 1.0 g of the nano adsorbent in 1 L of 40 ppm MO dye solution at 293, 303, and 313 K for time varying from 5 min to 1 day. The catalytic degradation of 40 ppm MO was approximately 67.6% at minutes, which increased to 84.9%, 91.5%, and 98.1%, 99.4% at 1 h, 2 h, and 3 h. In all these experiments, 5 mL of hydrogen peroxide was added. When only 1 mL of H_2_O_2_ was added to 40 ppm MO dye, the removal percentage was approximately 99.5%, which further decreased to 99.1 when 2 mL of H_2_O_2_ was added, and the removal percentage was almost the same, i.e., 99.1%, when 3 mL of H_2_O_2_ was added to 40 ppm MO dye. When 40 ppm MO dye+ 4 mL of hydrogen peroxide was added, then, after 3 h, the removal efficiency of MO dye was 98.88%. For all these conditions, results were achieved after 3 h. At 30 ppm MO dye, with 5 mL hydrogen peroxide, and after 3 h, the MO dye removal percentage was 98.7%. When the ppm was 20 and with 5 mL hydrogen peroxide, the MO dye removal efficiency was approximately 97.8% after 3 h. At 10 ppm, and along with 5 mL of H_2_O_2_, the MO dye removal percentage was approximately 95.4% after 3 h. Finally, at 5 ppm dye, along with 5 mL of H_2_O_2_, the removal was only 93.7% after 3 h (Da’na et al., 2018).

In the present study, approximately 36.72% of CR dye was removed after 60 min, while BG dye was removed up to 54.28% after 2 h, whereas the dye removal efficiency was more than 99% in the study led by Dana. However, in the current investigation, we performed experiments at neutral pH and at RT, while Dana et al. used hydrogen peroxide which felicitated the Fenton reaction. In addition to this, in the current study, we used a concentration of 50 ppm of BG and CR dye, while Dana et al. used 5–50 ppm. Moreover, we achieved about 54% BG dye removal after 2 h and 36.72% CR dye removal after 60 min, whereas Dana et al. carried out the investigation for 3 h. Nnaji et al. utilized the biochar from the *Dacryodes edulis* leaf for the removal of methylene blue dye up to 93% at pH 4, and it remained >70% even after three to four adsorption–desorption cycles (Nnaji et al., 2023).

Indriyani et al. synthesized a spherical-shaped, 9.94-nm-sized BiFeO_3_ nanocomposite by using leaf extracts of *Abelmoschus esculentus* L. Furthermore, they used the NPs for the photocatalytic degradation of MB dye, where approximately 94.04% degradation was achieved after 120 min under UV light (Indriyani et al., 2021). Yulizar et al. synthesized one-pot sol–gel-mediated V_2_O_5_–Fe_2_O_3_ nanocomposites of size 90 nm by using the leaf extracts of *Foeniculum vulgare*. The nanocomposite showed up to 94% removal of 4-nitrophenol from the aqueous solution (Yulizar et al., 2021b). Furthermore, Yulizar et al. synthesized SiO_2_/NiFe_2_O_4_ nanocomposites using the high-speed stirring (HSS) method using the leaf extract of *Kleinhovia hospita* L. and then used them for the reduction of 4-nitroaniline from aqueous solutions. They obtained about 95% of 4-nitroaniline reduction within 30 min (Yulizar et al., 2021a). Surya et al. synthesized 12–20-nm, spherical-shaped MgFe_2_O_4_ nanoparticles by using the leaf extract of *Cajanus cajan* (L.) Millsp and further studied their detailed optical, elemental, and optical properties (Surya et al., 2021).

### 4.10 Effect of pH on the remediation of CR and BG dyes

The pH of the dye solution has a significant impact on the adsorption capacity and rate of adsorption because it alters the stability, color intensity, and amount of dye molecules decomposed (Liang et al., 2021). The pH of the solution also governs the interaction between the charges of the adsorbent surface and ionized dye molecules (Nizam et al., 2021). The degree of protonation of the adsorbent surface groups varies with the acidic and alkaline pH. When the adsorbents are exposed to lower pH, the functional groups on the surface of the adsorbent become more protonated (Koohi et al., 2021).

**FIGURE 14:**
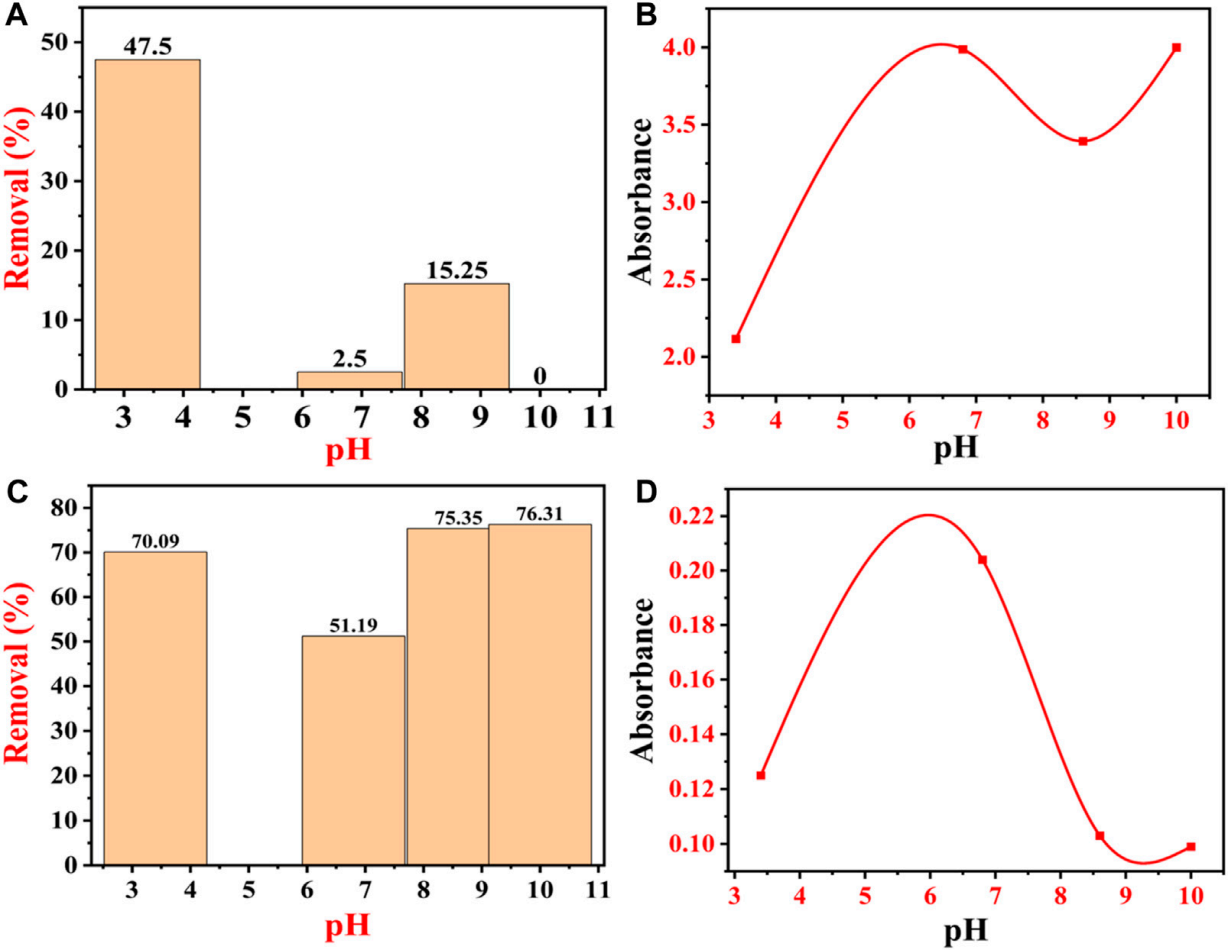
Effect of pH on the adsorption of dyes: **(A)** percentage removal of CR dye at different pHs; **(B)** absorbance peak of CR dye at different pHs; **(C)** percentage removal of BG dye at different pHs; and **(D)** absorbance peak of BG dye at different pHs.

Congo red (anionic) dye has a negative charge on its surface, while BG dye has a positive charge on the surface (Pandey et al., 2022). Both cationic and anionic dyes exhibit different mechanisms of dye adsorption behaviors under the same pH conditions. Under batch mode, the removal percentage of CR dye decreased from 47.75% to 0% with an increase in pH from 3.4 to 10. At pH 3.4, the CR dye removal percentage was 47.75, 2.5% at pH 6.8, 15.25% at 8.6, and 0% at pH 10. Figure 14A shows that the removal efficiency of CR dye decreases with the increase in pH, which could be related to the anionic nature of CR dye. At lower pH, i.e., 3.4, the removal efficiency of CR dye was maximum, i.e., 47.5%. At lower pH, IONPs carry a positive surface charge due to the protonation (H^+^), whereas CR dye molecules carry a negative charge due to an anionic sulfonate group (Hernández-Hernández et al., 2020). Moreover, at lower pH values, there are higher electrostatic attractions between negatively charged dye molecules and the positively charged surface of the IONPs, which enhances the dye removal. The phenolic groups present on the surface of IONPs from the plant extract are acidic in nature, and the possibility of alteration in the fabricated surface of IONPs is omitted at low pH (Mohamed et al., 2023). As the pH increased to 6.8, the removal efficiency of CR dye decreased to 2.5%, which might be de-protonated from the surface of the adsorbents. As the pH increased above 4, CR dye removal decreased at pH 6.8, which might be due to the increase in electrostatic repulsion between the negatively charged dye molecule and the negatively charged (-OH ions) surface of the IONPs (Kataria and Garg, 2017). In addition to this, there is a formation of an electrical double layer overlapping between the two charged electrons due to the appealing forces of London–van der Waals (VA) and the electrostatic repulsion (VR). At lower pH (acidic), there is an abundance of H^+^ ions in the aqueous solutions, due to which the surface of the adsorbent (IONPs) becomes positively charged, which, in turn, attracts the negatively charged CR dye molecules via electrostatic attraction (Wu, 2022). Furthermore, at pH 8.6, the removal efficiency of CR dye reached 15.25%, where there might be protonation on the surface of the adsorbent.

Finally, at pH 10, there was no adsorption of the CR dye molecule on the surface of IONPs; hence, there was 0% removal efficiency of CR dye, which might be due to the de-protonation from the surface of the adsorbents. At this stage, there is the possibility of repulsion between the CR dye molecules and the adsorbent molecules, thereby decreasing the removal efficiency of the CR dye. The removal percentage reached the lowest value at higher pH. Moreover, at pH 10, there is an abundance of -OH ions in the aqueous solutions of dye, due to which the negatively charged CR dye molecules get repelled from the surface of the negatively charged adsorbent (IONPs), so the adsorption ratio decreases (Pellenz et al., 2023).

The variations in the equilibrium of adsorption could be associated with the physicochemical properties and structure. So, for the effective and efficient removal of CR dye from IONPs, the pH should be near to acidic. So, the CR dye adsorption must be carried out either in weak acids or in a neutral environment (Nguyen et al., 2020).

Figure 14B shows the absorbance of dye from pH 3.4 to 10. CR dye is sensitive to pH and the transition of an azo group to a higher wavelength, which is caused by the protonation during the addition of HCl (Zainudin et al., 2023). When the acidic environment is too high, then the cationicity of the dye prevails, and due to protonation, two types of CR emerge, first, ammonium-rich and, second, azonium variety (Zhao et al., 2022). The ammonium-rich variety is mainly present in the fresh solutions, while the azonium variety is marked in the solution after 1 h, at which point the isoelectric point of CR is near 3 (Yermiyahu et al., 2007).

On the other hand, BG dyes are positively charged, so at lower pH, the electrostatic repulsion between BG dye molecules and the positively charged surface of the IONPs is very high, due to which there is a low removal percentage of BG dye from the aqueous solutions which leads to lesser dye removal (Tao et al., 2023). As the pH increases from acidic to neutral, the BG dye removal percentage also increases continuously, as shown in Figure 14C. Figure 14C shows that the percentage removal of BG dye by IONPs increases with the increase in the pH, i.e., 70.09% (pH 3.4), 51.09% (pH 6.8), 75.35% (pH 8.6), and 76.31% (pH 10). Between the neutral and basic pH, only a marginal (0.96%) increase in the dye adsorption was noticed, which might be due to the structural instability of BG at higher pH (Yermiyahu et al., 2007; Mane and Babu, 2011; Singh et al., 2022). At higher pH, the IONP surfaces adhere to negatively charged molecules, due to which the competition for H^+^ ions becomes less; hence, the BG dye removal becomes high (Joshi et al., 2019).

Another factor that plays a major role here is the pKa value; i.e., when the pH of the medium is lower than the pKa of BG, a large number of H^+^ ions compete for the free adsorption sites of IONPs with bulky BG dye molecules (Nizam et al., 2021; Csillag et al., 2023). As a result, the adsorption sites get saturated with the H^+^ ions instead of BG molecules, which results in a decrease in the amount of adsorption of BG dye molecules. The competition between H^+^ and BG molecule adsorption on the surface of IONPs disappears when the pH increases from acidic to neutral, as the concentration of H^+^ ions decreases. At neutral pH, practically all adsorbent surfaces are free for the adsorption of BG dye molecules (Ukkund et al., 2021). Figure 14C clearly shows that the electrostatic mechanism was not the only mechanism involved in BG dye adsorption. Figure 14D shows the absorbance of BG dye from pH 3.4 to 10. Table 2 and Table 3 show all the previous investigations for the removal of CR and BG dye by using IONPs, respectively.

**TABLE 2:** Previous investigations for the removal of CR dye by using IONPs and their composites.

Type of IONPs	Size of IONPs (nm)	Initial dye concentration (ppm)	Adsorption capacity (mg/g)	Removal %	Time of contact (min)	Reference
Incense stick ash	40–90			72%	60	Yadav et al. (2022)
Fe_3_O_4_/NiO	50	100		98.87%	90	Koohi et al. (2021)
Fe_3_O_4_ NPs functionalized with 1,2,4,5-benzenetetracarboxylic acid	20	20	630	97%	15	Chatterjee et al. (2020)
Fly ash@Fe_3_O_4_ mixture	Mean 3.34 µm (by DLS)	10–100	154 mg/g		20	Harja et al. (2022)
α-Fe_2_O_3_	200–500	50	139.86		60	Jia et al. (2015)
Fe_3_O_4_	200–500	50	84.96		120	
γ-Fe_2_O_3_	2,000–500	50	69.35		120	
γ-Fe_2_O_3_–Al_2_O_3_ phases		100	498		15	Mahapatra et al. (2013)
IONPs	12–28	50	305.59	33–47.5	60	Current investigation

**TABLE 3:** Comparative study of previous investigations for the removal of BG dye by using IONPs and their composites.

Type of IONPs	Size of IONPs (nm)	Initial dye concentration (ppm)	Adsorption capacity (mg/g)	Removal %	Time of contact (minutes)	Reference
Fe_core_−maghemite _shell_ (Fe−MM)	∼30 diameter	100	1,000			Ganguly and Ariya (2019)
Fe_3_O_4_@AC NPs	6–16	50	166.6	55.6%–91.8%	20–120	Joshi et al. (2019)
Fe_3_O_4_@SDBS@LDHs composites		50–120	329.1			ZHANG et al. (2017)
Magnetic barium phosphate composites			1,419.3 ± 10.0		0–60	Tao et al. (2023)
IONPs	12–28 nm	50	556.23	54.7	120	Current investigation

### 4.11 Mechanism of BG and CR dye removal by IONPs

The IONPs remediate dye molecules by adsorption under normal conditions, but in the presence of UV light, they act as a photocatalyst (Obaid and Halbus, 2023a; 2023b). So, during the adsorption of CR and BG dye by the IONPs, there is an involvement of numerous phenomena such as π–π stacking, electrostatic interaction, H-bonds, van der Waals forces, and coordination bonds (Kim and Choi, 2017; Litefti et al., 2019; Wang et al., 2022). There is a possibility of the involvement of a single process or a combination of processes in the adsorption of CR and BG dye molecules by the IONPs. There is a π–π stacking between the aromatic rings of CR dye molecules and π electrons of the IONPs, which includes charge transfer, scattering force, and polar electrostatic (Reza Masoodi et al., 2023). Second, there could be an electrostatic interaction between the net positive charges of IONPs pH < pH ZPC and the anionic structure of CR dye in the liquid media (Selvaraj et al., 2022). Third, there is a formation of H bonds between the existing –NH_2_ groups in CR dye and surplus –OH groups on the surface of the IONPs (Castellanos et al., 2019; Chatterjee et al., 2020). In addition to this, there could be the formation of coordination bonds between existing –NH_2_ groups and azo groups (–N = = N–) in CR dye with IONPs (Mezgebe and Mulugeta, 2022).

### 4.12 Regeneration study of IONPs

**FIGURE 15:**
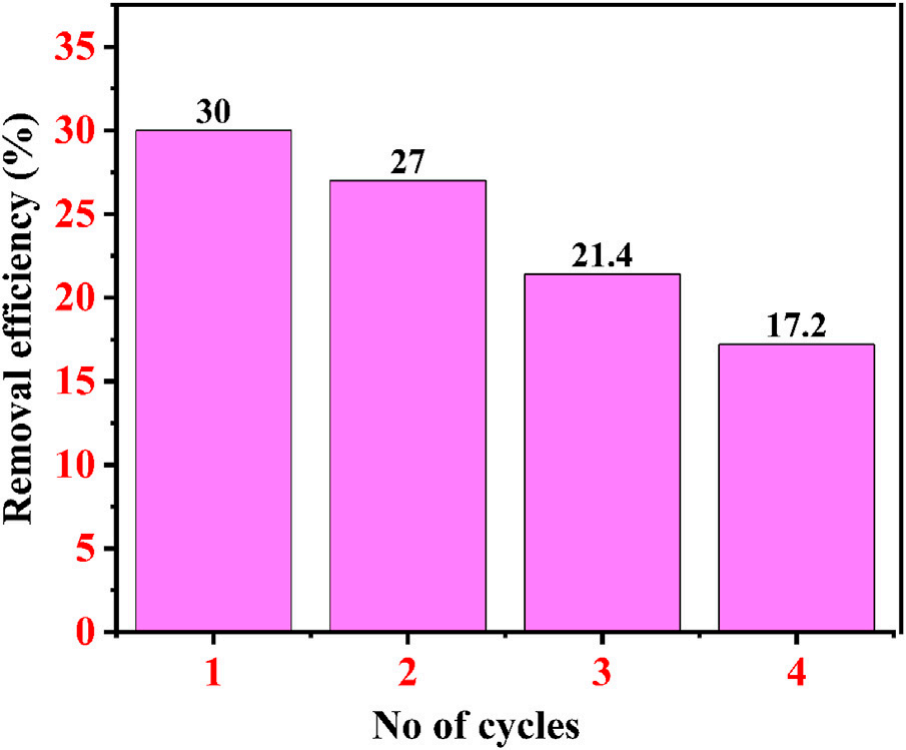
Regeneration study of IONPs for 50 ppm CR dye.

The regeneration study of an adsorbent decides the total cost of the dye effluent treatment for the industries. Here, in the first cycle, the removal efficiency was 30% after 60 min after the first cycle, while after the second cycle, the removal efficiency was 27% after 60 min. After the third cycle, the removal percentage of CR dye was approximately 21.4% after 60 min, and after the fourth cycle, it was only 17.2%, as shown in Figure 15. Choudhary et al. also performed a similar regeneration study for copper oxide NPs for the remediation of CR dye at 50 ppm, where after the first cycle, removal percentage was approximately 33.5%, 25.6 after the second cycle, 19.3% after the third cycle, and 16% after the fourth cycle (Choudhary et al., 2023a).

## 5 Conclusion

Iron oxide particles were successfully phytonanofabricated using the ethanolic extracts of *A. jacquemontii.* The various phytochemicals available in the plant play a major role as a capping agent which functionalizes the IONPs*.* The plant extract-assisted IONPs were synthesized by the chemical co-precipitation technique. The functionalized IONPs were analyzed using various analytical instruments, where the FTIR showed the bands reflecting phytochemicals from the plant on the surface of IONPs. The XRD showed an amorphous phase of IONPs with a peak at 33°–36°. The SEM showed that the size of IONPs was below 100 nm, with spherical to irregular shape. BG and CR red dye were removed up to 55% and 36% within 120 min and 30 min, respectively. The pH played an important role in the adsorption rate of the dyes, where acidic pH was suitable for the removal of CR dye and alkaline pH was suitable for the removal of BG dye. The regeneration study revealed a continuous decrease in the adsorption potential of the IONPs, and after the fourth cycle, its efficiency reached 17.2%. The current phyto-assisted technique could prove to be an eco-friendly and novel process for the phytonanofabrication of iron oxide particles and dye removal from wastewater.

## Data Availability

The original contributions presented in the study are included in the article/Supplementary Material; further inquiries can be directed to the corresponding authors.
